# Intelligent monitoring to predict atrial fibrillation (NOTE-AF): clinical study 1 for the ‘Health virtual twins for the personalised management of stroke related to atrial fibrillation (TARGET)’ project – a protocol for a prospective cohort analysis

**DOI:** 10.1136/bmjopen-2025-099658

**Published:** 2026-01-03

**Authors:** Hani Essa, Brian Johnston, Gregory Y.H. Lip, Sandra Ortega-Martorell, Karen Williams, Ingeborg D. Welters, TARGET Consortium, Sandra Ortega-Martorell

**Affiliations:** 1 Department of Cardiovascular and Metabolic Medicine, University of Liverpool, Liverpool, UK; 2 Royal Liverpool University Hospital, Liverpool, UK; 3 Liverpool Heart & Chest Hospital, Liverpool, UK; 4 University of Liverpool Faculty of Health and Life Sciences, Liverpool, UK; 5 Liverpool Centre for Cardiovascular Science, University of Liverpool, Liverpool, Merseyside, UK; 6 Danish Center for Health Services Research, Department of Clinical Medicine, Department of Teacher Education Aalborg, University College of Northern Denmark, Aalborg, North Denmark Region, Denmark; 7 Liverpool John Moores University, Liverpool, UK; 8 Critical Care Research, Royal Liverpool University Hospital, Liverpool, UK

**Keywords:** Patients, CARDIOLOGY, Machine Learning, Wearable Electronic Devices

## Abstract

**Introduction:**

Atrial Fibrillation (AF) is the most common arrhythmia worldwide affecting an estimated 5% of people over the age of 65 and is a leading cause of stroke and heart failure. Identification of patients at risk allows preventative measures and treatment before these complications occur. Conventional risk prediction models are static, do not have flexibility to incorporate dynamic risk factors and possess only modest predictive value. Artificial intelligence and machine learning-powered health virtual twin technology offer transformative methods for risk prediction and guiding clinical decisions.

**Methods and analysis:**

In this prospective observational study, 1200 patients will be recruited in two tertiary centres. Patients hospitalised with acute illnesses (sepsis, heart failure, respiratory failure, stroke or critical illness) and patients having undergone high-risk surgery (major vascular surgery, upper gastrointestinal surgery and emergency surgery) will be monitored with a patch-based remote wireless monitoring system for up to 14 days. Clinical and electrocardiographic data will be used for modelling the risk of new-onset AF. The primary outcome is episodes of AF >30 s and will be described as ratio of episodes/patient and as percentage of patients having episodes of AF. Secondary outcomes include 30-day and 90-day readmission rates and complications of AF.

The aim of this study is to generate data for the development and validation of health virtual twins predicting onset of AF in an at-risk population. The intelligent monitoring to predict atrial fibrillation (NOTE-AF) study is part of the TARGET project, a Horizon Europe funded programme which includes risk prediction, diagnosis and management of AF-related stroke (https://target-horizon.eu/).

**Ethics and dissemination:**

The study has received approval by the Health Research Authority and the National Research Ethics Service (REC reference 24/NW/0170, IRAS project ID: 342528) in the UK and has been registered on clinicaltrials.gov (NCT06600620). Results will be disseminated as outlined in the TARGET protocol to communicate project ideas, activities and results to diverse audiences.

**Trial registration number:**

NCT06600620.

**STRENGTHS AND LIMITATIONS OF THIS STUDY**

- This is a prospective, multi-centre observational study with predefined inclusion and exclusion criteria.
- The study uses continuous high-resolution recording of vital signs and ECG data, enabling detailed assessment of temporal physiological changes.
- Continuous single-lead ECG and vital signs monitoring will be performed using medical grade wireless technology.
- ECG data will be processed with automated, machine-learning–based analysis methods, which may reduce observer bias but are dependent on algorithm accuracy and quality of training data.
- This observational study will be conducted alongside routine clinical care. No direct changes in clinical management will occur based on the observations obtained.

## Introduction

 Atrial fibrillation (AF) is the most common cardiac arrhythmia worldwide affecting an estimated 5% of people over the age of 65 and is a leading cause of stroke and heart failure.^1^ AF is a heterogeneous condition provoked by various underlying pathophysiological disorders and is frequently first diagnosed in acute illness or during and after surgical interventions. The main risk factors include but are not limited to diabetes mellitus, advancing age, hypertension and coronary artery disease.^1^ In the UK, the estimated healthcare costs from AF in 2020 ranged from £1435 to £2548 million.^2^

AF confers a fivefold increased risk of stroke and is ultimately implicated in roughly 20% of all strokes.^3^ In Europe, stroke is the second most common cause of death and a leading cause of disability with an estimated annual cost of €45 billion.^2^ These numbers are expected to increase due to an ageing population and improving survival rates.^4^ The pathophysiology of AF-related stroke (AFRS) is associated with severe neurological deficits due to larger clots, infarction of substantial cerebral areas and haemorrhagic transformation, a complication that significantly worsens prognosis. Functional recovery from AFRS is often unsatisfactory, leading to longer hospital stays, severe disability and high mortality.^5^

Traditionally, AFRS is associated with various stroke risk factors, whereby the more common and validated ones have been used to formulate clinical stroke risk stratification scores, such as CHA2DS2-VASc ([congestive heart failure, hypertension, age ≥ 75 (doubled), diabetes, previous stroke/transient ischaemic attack/thromboembolism (doubled), vascular disease, age: 65–74, sex (female)])/CHA2DS2-VA.^6^ However, these are limited by their modest predictive performance.^7^ Risk factors, such as renal impairment, inflammation, atrial remodelling and cardiac and cerebrovascular biomarkers, are not considered in these routine risk scores, which are often based on assessment at one time point and outcomes occurring many years later. However, risk is not static but dynamic in nature, changing with ageing, development of comorbidities and acute events.

Artificial intelligence (AI) solutions are increasingly used in clinical practice for the prediction and detection of diseases and events, optimisation of treatments, etc significantly outperforming more traditional techniques based on signal processing.^8 9^ Personalised AI-based prognostication and decision support tools taking account of the patient individual characteristics, medical and health status history will enhance risk prediction, leading to ultra-early AF identification, guide treatment decisions and prognosticate outcomes. The inclusion of biomarkers related to cardiac damage and automated analysis of ECG characteristics adds further pathophysiological information to the AI modelling. With rapid changes in such biomarkers and vital signs, a dynamic and personalised risk assessment approach, accounting for individual pathophysiological risk factors, is required to reliably predict outcomes. Accounting for dynamic changes in risk factors may improve clinical risk prediction.^10 11^

Patients admitted to hospital either for elective surgery or because they are acutely unwell represent an opportunity to obtain continuous monitoring data. AF is common in patients suffering from acute conditions such as infection, stroke, acute heart failure, acute respiratory failure or following major surgery, in particular, vascular and upper gastrointestinal surgery.^12^,^14^ Many of these episodes occur in patients without a history of AF. Episodes of AF during acute conditions represent a clinical deterioration in acutely unwell patients and lead to increased short and long-term morbidity and mortality.^15^

Postoperative AF (POAF) is the most frequently reported cardiac arrhythmia following non-cardiac surgery. The incidence of POAF is highly variable but estimated to be between 0.5% and 15% in all non-cardiac procedures.^16^ Following upper gastrointestinal surgery, a particularly high incidence of POAF has been observed, with up to 45.5% of patients undergoing oesophageal resections displaying episodes of AF postoperatively,^17 18^ and this is associated with increased 30-day mortality, stroke and hospitalisation. In 18% of cases, patients suffer further episodes of AF within 1 year with 27% of these patients progressing to paroxysmal or persistent AF.^17^

AF is conventionally diagnosed by 12-lead ECG recordings demonstrating irregular R-R intervals (The interval between consecutive R-wave peaks on the ECG) without recognisable p-wave activity with a duration of >30 s. Recordings from implantable and patch-based cardiac monitoring devices suggest that many patients experience episodes of AF which are not diagnosed clinically.^19^ Such episodes are not benign entities: Multiple systematic reviews have demonstrated a fivefold increased risk of developing clinically relevant forms of AF and a 2–3-fold increased risk of stroke and thromboembolic events.^20 21^

The technological advances of medical devices for vital signs measurements have led to the development of wireless continuous vital sign monitoring systems (wCVSM) with real-time data presentation to healthcare professionals. These systems can identify vital sign deviations that are not found with intermittent manual monitoring,^22 23^ potentially identifying patients at risk earlier. In this study, we will use continuous ECG monitoring integrated into wCVSM systems to identify episodes of AF >30 s, independent of their clinical recognition.

The intelligent monitoring to predict atrial fibrillation (NOTE-AF) study will take place in two large tertiary referral centres at the Liverpool University Hospitals NHS Foundation Trust, which are part of the University Hospitals of Liverpool Group. The data obtained in NOTE-AF will feed into a large European Union (EU) funded project (TARGET: https://target-horizon.eu) which aims to develop multi-scale and multi-organ, dynamic, and interoperable virtual twins of AF patients to accelerate translational research, enabling personalised and cost-effective management.^24 25^ Within TARGET, personalised virtual twin models for dynamic risk prediction of AF and AFRS that consider individual patient characteristics and medical and health status history will be developed. TARGET is a research programme led by a consortium of 19 European partners aiming to reshape the risk prediction, diagnosis and management of AF and AFRS, and accelerate the translation of research into practical applications. Personalised AI-based prognostication and decision support tools taking account of patient individual characteristics will enhance risk prediction and lead to early identification of AF and personalised treatment decisions. The two work packages (WP) comprised in the NOTE-AF study are linked to the TARGET project, with WP1 representing the observational part of NOTE-AF, which is described in the manuscript.

## Methods

NOTE-AF is a prospective, multi-site, observational study assessing the incidence and duration of clinical and subclinical episodes of AF in cohorts of postoperative and acutely unwell hospitalised patients. The study consists of two WP, a prospective observational study (WP1) and a qualitative feasibility study (WP2) and addresses the secondary objectives and outcomes listed below. The protocol for WP2 will be summarised elsewhere. Patients will be recruited within 72 hours of admission and undergo up to 14 days of total ECG monitoring. Patients will be recruited over a 4-year period starting in October 2024. Clinical and ECG data obtained in NOTE-AF will then be used to construct virtual twins to predict clinical and subclinical episodes of AF as part of the TARGET programme (https://target-horizon.eu/the-project/).

### Study population, inclusion and exclusion criteria

In the UK, the estimated prevalence of AF is 3% in the general adult population^26^ and increases to 10%–15% in acutely hospitalised and critically ill patients. The incidence of subclinical AF (SCAF) is likely to be higher. We presume an incidence of at least 5% in our cohort of acutely unwell hospitalised patients and therefore expect >100 cases in 1200 patients recruited over a 4-year period.^27^ The inclusion and exclusion criteria are detailed below.

#### Inclusion criteria for enrolment

Adult patients ≥50 years undergoing major upper gastrointestinal or vascular surgery or admitted to hospital with at least one of the following acute conditions associated with an estimated risk of developing episodes of AF >5%:

- Acute respiratory failure.
- Any acute illness referred or admitted for higher level of care (organ support).^28^
- Patients with acute stroke.
- Patients with acute heart failure.
- Patients admitted to the emergency department with clinical suspicion of severe acute infection or sepsis requiring hospitalisation.^29^

#### Exclusion criteria for enrolment

- Any previous history of atrial fibrillation or atrial flutter.
- Paced cardiac rhythm.
- Inability to obtain consent (patient and/or consultees decline study participation).
- Allergy to plaster or silicone.
- Expected hospital stay <48 hours.

#### Criteria for Premature Withdrawal

- Withdrawal of consent.
- Intolerance of monitoring patch.
- Persisting skin irritation at patch site.

A copy of the participant consent form and patient information leaflet, including a consent form for patients who have regained capacity, is available in the online supplemental appendices.

### Study interventions

#### Continuous patch-based wireless ECG monitoring

**Figure 1:**
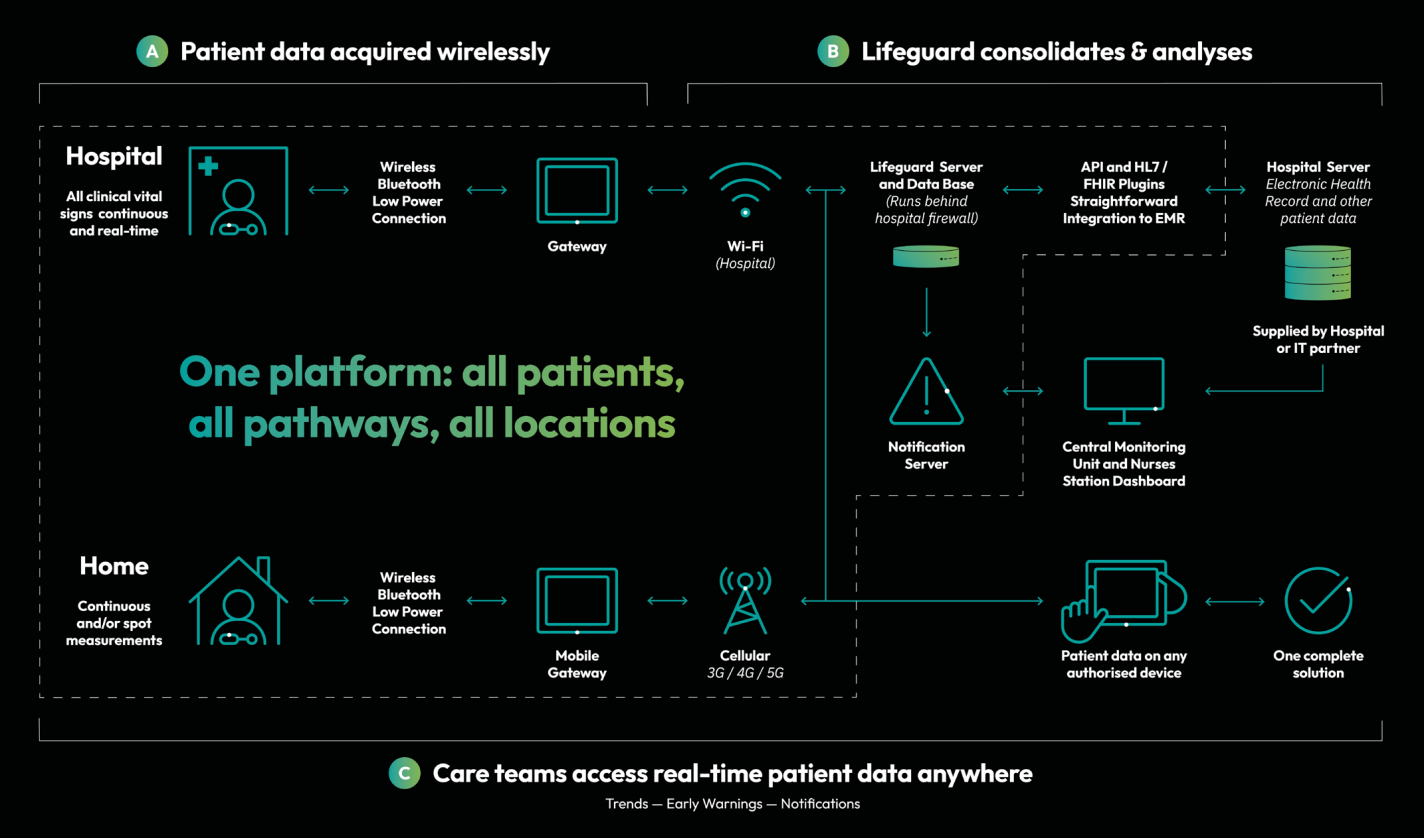
Isansys platform and connection between system and hospital. Copyright Isansys (used with permission). API = application programming interface, HL7 = Health Level Seven, FHIR = Fast Healthcare Interoperability Resource, EMR = electronic medical records. 3G = Third generation, 4G = Fourth Generation, 5G = Fifth Generation

Patients who fit the inclusion criteria and provide informed consent will undergo ECG monitoring using the Isansys Lifetouch sensor combined with the Patient Status Engine (Isansys Lifecare, Oxfordshire, UK), a Food and Drug Administration (FDA) approved and conformité européenne (CE marked) marked monitoring system. An ECG patch with two electrodes continuously records a single lead ECG, heart rate and respiratory rate.^30^ Data from the Isansys LifeTouch sensor will be automatically transmitted via Bluetooth to a password-protected tablet device kept next to the patient’s hospital bed. Data are displayed on the tablet (Patient Gateway) in real time and securely stored on a server (figure 1). The device records the ECG in a visual format and allows the end user to access the raw data. This generates a record of the amplitude of the ECG with a time stamp at a rate of 100 recordings per second (100 Hz) and allows reconstruction of an ECG from a comma-separated values (CSV) file. ECG analysis will occur using traditional clinician interpretation and Poincaré plots, automated image analysis and convoluted neuronal networks.^8^ ECG monitoring will continue for 14 days or until hospital discharge, whichever occurs first. All patients will continue to receive standard routine care in addition to wCVSM. Standard clinical treatment will be provided in line with the organisation’s policies for monitoring of vital signs. In case of clinical deterioration, standard treatment pathways will be followed.

### Baseline and admission data

Baseline and admission data are displayed in table 1.

**Table 1:** Baseline and admission data collected in clinical record form

Type of data collected	Data points/variables
Demographics	Date of hospital admission, age, gender, study arm, height, weight, ethnicity, medical history, Charlson Comorbidity Index, Frailty Score
Admission/preadmission ECG data	Bundle branch block, p-wave duration, PR interval
ICU (Intensive care unit) data	Date of ICU admission, type of organ support, type of ICU admission, type of sepsis, ICU admission diagnosis, disease severity scores, 24 hours fluid balance
Perioperative data	Type, urgency, duration and severity of surgery, indication for surgery, risk classification
Cerebrovascular events	Type of stroke, treatment for stroke, NIH Stroke Scale/Score, CT findings
Presence of heart failure	Aetiology, symptoms, treatment, type and severity of heart failure,
Admission medication	Antihypertensives, anticoagulation, anti-arrhythmic
Admission blood results	Full blood count, electrolytes, renal and liver function tests, coagulation
Biomarkers	C-reactive protein, troponin, lactate, pro-BNP (B-type natriuretic peptide)
Admission vital signs	Blood pressure, heart rate, temperature, respiratory rate, oxygen saturation
Arrhythmic events	Occurrence, time of onset, treatment of AF, bradycardia, tachycardia, non-AF arrhythmias
Cardiovascular complications	Cardiac arrest, myocardial ischaemia, hypotension
Respiratory data	Hypoxia, hypercapnia, type of ventilation, type of oxygen therapy
Outcomes	Hospital mortality, 90-day mortality, length of hospital stay, length of ICU stay

AF, atrial fibrillation; NIH, National Institute of Health.

### Blood sampling

Surplus serum blood samples will be collected on the day of recruitment (day 1) and each day until day 7 for up to a maximum of 5 blood samples per patient. Pseudonymised samples will be centrifuged, plasma or serum will be aliquoted, frozen and stored at −80°C for subsequent analysis. Serum samples will be used to determine cardiac biomarkers, including but not limited to troponins, natriuretic peptides and inflammatory markers.

### End of Study Definition

The expected study duration is 48 months, including setup, recruitment, 6 months follow-up period for data cleaning and analysis and write-up of results. The study will close after data are analysed and a final report has been submitted to the funder.

### Ethical conduct

This study is conducted in compliance with international ethical and scientific quality standards, known as good clinical practice (GCP) and with the principles of the Declaration of Helsinki. This study was approved by the North West – Haydock Research Ethics Committee and the health research authority (IRAS (Integrated Research Application System) project ID 342528, REC (Research Ethics Committee) reference 24/NW/0170).

### Primary research objective

To determine the incidence of clinical and subclinical episodes of AF in acutely unwell patients and to generate data for the development and validation of virtual twins and clinical decision support tools.

### Secondary research objective

To determine patient acceptability and usability for healthcare professionals of a novel remote monitoring device with automated alert function.

### Primary Outcome

The incidence of episodes of AF lasting >30 s in acutely unwell and postoperative patients recorded as part of standard clinical care through extended remote wireless patch-based monitoring with or without the use of an alert system (Application).

### Secondary outcomes

- Length of stay.
- Hospital readmissions within 90 days.
- Hospital and 90-day mortality.
- Recurrence of AF episodes,
- Time spent in AF.
- Number of AF episodes.
- Complications of AF, for example, stroke, thromboembolic events.
- High sensitivity troponin concentrations in patients with AF episodes.
- Echocardiographic changes in patients with AF episodes.
- MAUQ (mHealth app usability questionnaire) score.
- Percentage change of troponin concentrations in patients with and without episodes of AF.
- Time (in hours) wCVSM is attached.
- Number of cardiovascular alerts.
- Number of non-cardiovascular alerts.
- Number of alerts reflecting clinical changes.
- Number of alerts reflecting artefacts or non-clinical events.
- RWMA (regional wall motion abnormality) score, atrial size and volume, left ventricular strain rate, standard echocardiographic measurements as per BSE (British Society of Echocardiography) recommendations.
- Change in inflammatory markers white cell count, C-reactive protein and procalcitonin over time.

Usability and acceptability data will be collected to inform the design of a separate implementation study or a process evaluation.

### Statistical analysis

#### Sample size considerations

The primary aim of this study is to generate data for the development of health virtual twins as part of the EU Horizon 2023 TARGET project. We will therefore collect a convenience sample of 1200 patients with a risk of developing episodes of AF of at least 5%. Given evidence from the literature that the incidence of subclinical episodes that remain undetected may be >30%,^27^ we expect that in a cohort of 1200 patients >100 patients will be diagnosed with episodes of AF of at least 30 s.

#### Statistical methods

The primary aim of the study is to obtain data for the generation of dynamic risk prediction tools and virtual health twins. The methodology of developing and refining the virtual health twins will be described elsewhere. In line with the primary objective, conventional statistical analysis will be limited and conducted to support the development of virtual health twins by description of AF episodes and is described as follows:

The primary outcome (incidence of AF and SCAF episodes >30 s in acute illness) will be described as ratio of episodes/patient and as percentage of patients having episodes of AF. We will also analyse time spent in AF as a percentage of time monitoring was in place, distribution pattern of AF, duration of AF episodes, occurrence and frequency of premature atrial complexes as precursors of AF episodes. We will use conventional logistic regression models to establish the correlation and weight between risk factors for AF (eg, age, cardiovascular comorbidities, organ dysfunction and support, blood pressure, infection status etc) and occurrence of AF. Outcomes will be compared in univariate analyses, using parametric or non-parametric test depending on sample distribution. Descriptive statistics will be used to describe demographics, frequency of alerts and blood results in patients with and without episodes of AF. Group comparisons will include parametric (T-Test for independent samples) and non-parametric tests (Mann-Whitney-U-Test), depending on sample distributions. For categorical data, Fisher’s exact test will be used to examine associations between variables. Monotonic relationships between two numerical variables will be defined using Spearman’s rank correlation coefficient. Where applicable, univariate analysis will be followed by multivariate logistic regression analysis to identify risk factors for the development of AF episodes postoperatively. Cochran-Mantel-Haenszel test (CMH) will be used for the analysis of stratified categorical data, using occurrence of AF episodes >30 s as the binary outcome.

Further statistical analysis will depend on the modelling results obtained as part of the specific objective “To develop and validate personalised virtual twin models for the dynamic risk prediction of AF and AF-related stroke” of the TARGET project (https://target-horizon.eu/the-project/). The methodology of the virtual health twin development will be published separately. Existing retrospective data from publicly available healthcare data repositories and from TARGET consortium partner organisations will be used to develop a centralised data platform for modelling, employing state-of-the-art data integration and harmonisation techniques. We will combine established risk factors with comorbidities, imaging and biomarkers for all stages of the AFRS pathway. The resulting personalised models will advance clinical care and enable dynamic, longitudinal monitoring. Validation of the models obtained will occur via in silico trial techniques to accelerate the translation of the virtual twin technology and to generate evidence for refinement and further development of the twin technology.^25^ The in silico trials will be developed as part of TARGET and methodology will be described elsewhere.

### Data Collection and Management

In line with requirements for European Horizon funded projects, a comprehensive Data Management Plan (DMP) will ensure systematic organisation and easy accessibility of data and compliance with legal, ethical and EU requirements. The DMP will allow easy accessibility of data and support consistency and quality of data handling through standardised procedures. Additionally, it outlines strategies for data storage, backup and long-term preservation to prevent data loss and ensure future usability. The use of Electronic Patient Health Records is part of standard care at the recruiting site to document routine patient data and will allow extraction of standard data, including length of stay, admission diagnosis, date, time and reason for readmission, new diagnoses and clinically relevant cardiovascular events.

### Patient and public involvement

Patients and the public involvement forms a separate work package of the EU HORIZON 2023 TARGET programme.

### Adverse events

For this observational study, we are not expecting any adverse events. However, our clinical records form allows the input of any adverse events.

## Discussion and conclusions

Limited information is available in the literature regarding incidence, risk factors, diagnosis and treatment of AF in the acutely unwell hospitalised population. Unless continuous monitoring is applied or clinical symptoms occur, transient episodes, particularly those of very short duration, will remain undetected, leading to a gross underestimation of the actual incidence of AF episodes.^22 31^ Using remote wireless continuous monitoring, we will clarify the incidence of clinical and subclinical episodes of AF in acutely unwell and high-risk perioperative patients. ECG data generated at a sampling rate of at least 100 Hz will then, in combination with clinical data and data from other European centres, be fed into TARGET for the development of virtual health twins comprising integrated, multi-scale computational models for personalised risk prediction, optimised management and treatment.

Where new episodes of AF are detected as part of standard clinical care, treatment will follow national and international guidelines.^32^,^34^ However, new-onset AF (NOAF) in acute care remains a clinical challenge, as subclinical episodes usually remain undiagnosed, follow-up for patients with resolving AF during recovery from acute illness is not well described and current treatment recommendations have been derived from general patient cohorts without focus on acute triggers and bleeding risk in acute clinical situations. Therefore, acute care clinicians extrapolate from populations who differ with regard to aetiology, pathophysiology and commonly prescribed therapies when treating acutely unwell patients with AF.^35^

It is well documented that in acutely unwell patients, the majority of cases of NOAF occur within the first 3 days of admission. This is because the burden of acute systemic stressors/triggers (hypoxia, electrolyte disturbance, cytokine surges and sympathetic activation) is often highest in the early phase of the disease. Prior work on patients suffering from septic shock demonstrated that episodes of NOAF usually occur prior to day 5.^30^ Extending the monitoring period to 14 days will allow us to collect information about recurrence and duration of episodes of NOAF in acute illness. Previous literature suggests that in patients hospitalised for non-cardiac surgery or medical illness who had transient NOAF, recurrence occurs in one third of patients within 1 year.^36^ Similarly, recurrence of AF episodes in ICU (Intensive Care Unit) patients who are treated successfully for their first episode is also common.^13 37^

While all patients will undergo patch-based cardiac monitoring, a small number of patients (20) will undergo continuous vital signs monitoring (respiratory rate, saturations and blood pressure). Based on these vital signs, alerts will be created that will be sent to nursing staff. In this qualitative data collection exercise (WP2), we will monitor behavioural responses and views of healthcare professionals and the correlation between these alerts and routine observations. We will use the MAUQ questionnaire,^38^ a validated and structured instrument designed specifically for evaluating mHealth systems.

Recent advances in continuous monitoring devices, including patch-based wireless technology, allow close monitoring of heart rhythm and vital signs over prolonged periods without impact on patient mobility and comfort. Integrated repeat sampling techniques can identify short, otherwise undiagnosed arrhythmic episodes reliably in patients compared with intermittent vital signs monitoring as part of standard care.^22^

In non-stroke patients, asymptomatic AF will be of uncertain causal relation to the index hospitalisation and therefore, the role of anticoagulation remains tenuous. The benefits of anticoagulation for the prevention of stroke in acutely ill patients with NOAF have been insufficiently studied. The burden of AF is usually low, with most patients converting to sinus rhythm as acute triggers resolve. Only in the embolic stroke of undetermined source (ESUS) population, it is standard practice to perform prolonged ECG monitoring and start anticoagulation once AF is found.^39^,^41^

In summary, the NOTE-AF study will apply continuous cardiac monitoring of acutely unwell patients to understand and describe the incidence of clinical and subclinical AF. The data from NOTE-AF will be fed into TARGET to produce AI-based computational models for personalised risk prediction. These virtual health twins will offer dynamic real-time decision support for clinicians in acute and perioperative care by integrating physiologic, environmental and healthcare data into machine learning algorithms and generative models to accelerate and facilitate personalised interventions and prevent adverse outcomes of AF.

## Recruitment to date

NOTE-AF started recruitment on 29 October 2024, and to date, 85 patients have been recruited. Recruitment was paused following the bankruptcy of the original equipment supplier (Isansys). Two alternative suppliers (SmartCardia and Checkpoint Cardio) have since been identified, requiring new procurement and IT (information Technology) integrations. A major ethics amendment has been submitted and approved to enable use of these devices. Recruitment has now restarted. The overall trial design and objectives remain unchanged.

## Supplementary material

10.1136/bmjopen-2025-099658 online supplemental file 1

10.1136/bmjopen-2025-099658 online supplemental file 2

10.1136/bmjopen-2025-099658 online supplemental file 3

10.1136/bmjopen-2025-099658 online supplemental file 4
