# Supplementary material for: Intelligent monitoring to predict atrial fibrillation (NOTE-AF): clinical study 1 for the ‘Health virtual twins for the personalised management of stroke related to atrial fibrillation (TARGET)’ project – a protocol for a prospective cohort analysis
Source: BMJ Open. 2026 Jan 3;16(1):e099658. doi: 10.1136/bmjopen-2025-099658 (PMC12766758; doi:10.1136/bmjopen-2025-099658)
Supplement: online supplemental file 1 [file bmjopen-16-1-s001.pdf]

PARTICIPANT INFORMATION SHEET

STUDY TITLE:

Intelligent Monitoring to Predict Atrial Fibrillation [NOTE-AF]: Clinical study 1 for the "Health virtual twins for the personalised management of stroke related to atrial fibrillation (TARGET)" project.

NAME OF RESEARCHERS: Professor Ingeborg Welters, Dr Hani Essa and Dr Brian Johnston

Covering statement, participant information leaflet.

IRAS ID: 342528

You are being invited to take part in a research study that is being carried out at Liverpool University Hospitals NHS Foundation Trust. The study is called NOTE-AF. NOTE-AF is hoping to find out how often patients have irregular heartbeats and use the information to highlight at risk patients and improve care in the future using modern computer technology. We aim to recruit 1200 patients from Liverpool to take part in this initiative.

NOTE-AF is part of a large research project called "TARGET", which is funded by the European Union (https://target-horizon.eu). TARGET aims to improve the management of a particular form of irregular heartbeat called "Atrial Fibrillation" (AF) and its most severe complication, AF-related stroke (AFRS). Despite extensive research to prevent strokes, we do not fully understand the links between AF and stroke, the long-term risks, and the complications. The main aim of the TARGET is to develop a computer tool, also called "virtual twin", so that doctors can make better decisions how to treat atrial fibrillation, a common form of irregular heartbeat. Within the TARGET project, several clinical studies are conducted to collect data for the development of these virtual twins. In the NOTE-AF study we wish to collect data such as heart rate, oxygen levels and blood pressure using an approved and licensed wireless monitoring system.

Before you decide whether you would like to take part it is important for you to understand why the research is being done and what it involves. Please take time to read the following information carefully and feel free to ask us if you would like more information or if there is anything that you do not understand. Please also feel free to discuss this with your friends,

relatives, and GP if you wish. We would like to stress that you do not have to accept this invitation and should only agree to take part if you want to.

Taking part in this study is entirely voluntary. Your treatment will not be affected if you decide not to take part.

Thank you for your time in considering this request.

WHAT IS THE PURPOSE OF THE STUDY

Atrial fibrillation (AF) is a condition where the heartbeat becomes fast and irregular. It is the most common type of heart rhythm problem and left untreated can cause serious complications such as blood clots or a stroke.
AF can be a complication for patients admitted to hospital with infection, stroke, heart failure, acute respiratory failure, or following major surgery.
Patients who develop AF may have no symptoms at all and for some patients the irregular heartbeat comes and goes meaning that it can be missed if heart rate checks are only done at set times during the day.
It is therefore important to detect AF early, and identify which patients are at increased risk of developing it. Using modern computer technology ("artificial intelligence"), computer scientists can develop tools that can help inform doctors about the risk of each patient developing AF. This would allow doctors to take measures to prevent AF or to request further investigations of the heart to better understand the cause. In this project, we wish to collect data to develop such a tool, also called a "virtual twin". A virtual twin is a computer model that receives real-time patient data, analyses them, and sends information or suggestions for treatment back to doctors, nurses, or patients. In short, a virtual twin is a dynamic computer model which supports healthcare professionals but also patients in making informed treatment decisions. Testing of this virtual twin in patients will be part of a follow-up study, this project only serves to obtain the data necessary to develop the tool.
NOTE-AF will use a wireless patch that will record your heart rhythm continuously. You will also be attached to a blood pressure device which measures your blood pressure at regular intervals. We will also measure your oxygen level with a finger clip.
Some wards will take part in a second part of the study called WARD 247. In WARD 247 the nurses looking after you will get alerts to a special mobile phone if any abnormal readings are detected. In total, 25 patients will take part in WARD 247. If you are being treated on a ward where the WARD 247 system is in use, you may be asked to fill in questionnaires so that we can better understand how to improve the monitoring system and the way we use the mobile application. The questionnaire will ask about the type of monitoring you were wearing and how you felt about when it was used. Filling in questionnaires can cause distress, however, in this case it is unlikely that any sensitive issues are addressed in the questions we ask. Participation in the WARD 247 survey and filling in the questionnaire is optional. If you do not wish to do so, you can still take part in NOTE-AF.

WHY HAVE I BEEN INVITED TO PARTICIPATE?

You have been invited to take part in this research study because you are over 50 years of age and have been admitted to hospital for one or more of the reasons below.

- Acute heart failure
- Sepsis or infection
- Surgery on your stomach or oesophagus (food pipe)
- Vascular surgery (operations on large blood vessels)
- Acute respiratory failure
- Acute stroke
- You are so unwell that you have been referred for treatment in the Intensive Care Unit

You should not take part if you already have AF, have a pacemaker or any known allergy to plasters or silicone.

DO I HAVE TO TAKE PART?

No. Joining this research study is entirely voluntary. If you are interested in taking part, you will be given this information sheet to keep and asked to sign a consent form.

You are free to withdraw at any time and without giving a reason. A decision to withdraw at any time, or a decision not to take part, will not affect the standard of care you receive and will have no influence on your future treatment. The doctors caring for you will give you the same treatment regardless of whether you participate in this study or not.

WHAT WOULD TAKING PART INVOLVE?

All patients that agree to take part in the study will have heart rate, breathing rate, blood pressure and oxygen levels measured as per the normal hospital practice.

As part of the study, we will ask you to also wear a wireless patch, attached to the chest by two sticky electrode dots. This device will measure your heart rate and rhythm and download the results onto a computer hard drive for analysis. We will also record your blood pressure more often than we would do normally and capture your oxygen levels with a finger clip.
If you have been admitted for surgery the monitoring will be attached after your surgery.
If you have been admitted as an emergency the monitoring will be attached soon after you have agreed to be part of the study.
The monitoring will continue for 7 days, unless you go home before then or decide you no longer want to be part of the study.
As part of the research study, we will also record other routinely collected information including:

- Demographic data (age, weight, height, and gender)
- Your medical history

- Your usual medicines
- Details of any surgery
- Reasons for any Intensive Care Unit admission
- The results of routine blood tests or investigations
- Medications administered whilst in hospital and any medications for abnormal heart rhythms.
- Your total length of hospital stay.

All information will be collected by a trained member of the research team. All information will be collected by accessing routinely available results in your electronic medical records.

Leftover blood samples from routine blood testing will be collected between days 1 and 7 and within 24hrs of hospital discharge. These blood samples will be stored in freezers within the hospital or transferred to the Liverpool University Biobank for long-term storage. We may wish to use these samples for research in future projects, but only if you give consent to do so.

If we approach you to take part in the WARD 247 study, we may also ask you to complete questionnaires on how you have found the extra monitoring and which type of monitoring you have been wearing. If you are participating in NOTE-AF on a ward that is part of WARD 247, alerts for any abnormal readings will be sent to the ward nurse on a dedicated study mobile phone. As part of the study, we will also ask the nurses looking after you how useful the alerts are in improving your care. Filling in the questionnaire is an optional part of NOTE-AF. You can still take part in the study if you do not wish to take in WARD-247.

If we find any abnormal values which may need to be investigated further, we will send a letter to your GP, informing them that you have been enrolled in this research study. We will seek your consent before doing so. We may also wish to ask your GP for information from your medical records if we do not have them on our hospital records.

All patients will be followed up at 90 days, to see if there have been any changes to their health. The research team will look at your health records, there will be no need to come into the hospital.

WHAT IS THE MONITORING EQUIPMENT, WHAT DO I NEED TO KNOW?
We will monitor your vital signs, including heart rate, blood pressure, respiratory rate, and temperature, using a cutting-edge technology called the Patient Status Engine (PSE) provided by a company called Isansys Lifecare Limited. The system wirelessly transmits your vital signs data to healthcare professionals in real-time and can detect changes in your health status early, allowing for timely interventions if necessary.

WHAT ARE THE POSSIBLE BENEFITS OF TAKING PART?

It is unlikely that there will be any specific benefits for patients taking part in this research study. Even though there will be continuous monitoring of your heart rate and rhythm for 7 days, it is important that you know that the recordings will not be monitored all the time by the research team. However, when reviewing the recordings and when

any problems with your heart rate are found on the recordings, the research team will notify your hospital consultant or GP to arrange for further investigations.

If you are part of WARD 247 alerts for any abnormal readings will be sent to the ward nurse on a dedicated study mobile phone. The nurse may respond quicker to any abnormal findings than with conventional monitoring. However, we do not know yet if this will regularly happen.

All care will be directed by the doctors looking after you.

The study is most likely to benefit patients in the future, once the virtual twin tool has been developed, as it will provide more information on who is at risk of developing AF.

WHAT ARE THE POSSIBLE DISADVANTAGES AND RISKS OF TAKING PART?

It is highly unlikely that you will come to any harm from taking part in this research study. The research team will not have any influence on your treatment. All care will be directed by the doctors looking after you. The monitoring devices we intend to use have been safety tested, are already in use in the NHS and will not interfere with the routine monitoring that is used in the hospital.

WHAT HAPPENS IF I DON'T WANT TO CARRY ON WITH THE STUDY?

You are free to withdraw your consent to participate at any time and without giving a reason. This will not affect the standard of care you receive. No further samples or data will be collected upon the point of withdrawal but those already held will be retained. Your study doctor can take you out of the study at any time if it is in your best medical interests to stop your participation. Please approach a member of the research team (telephone 0151 706 2410, email ITUresearchteam@rlbuht.nhs.uk), if you do not wish to take part in the study anymore.

STORAGE OF BLOOD SAMPLES.

The samples taken will be stored in anonymised format at the Royal Liverpool University Hospital. At a later stage and if you agree, we will transfer the samples to the Liverpool University Biobank. Samples are stored according to appropriate regulations. We would like to store your samples after the study has finished, however, if you do not want this, you can still participate in the study. Any samples will be disposed of when the study analyses are completed. The reason we want to store samples is that if new information or techniques are discovered in the future this will allow us to use the samples stored to investigate if this new information is important.

WILL TAKING PART AND MY DETAILS BE KEPT CONFIDENTIAL?

Any information that is collected about you during the course of the research will be kept strictly confidential. Any information about you that leaves the hospital will have any identifying information removed from it. All procedures for handling, processing storage and destruction of data are compliant with the Data Protection Act 2018 and GDPR guidelines. The NOTE-AF study is part of a large European Research project. This requires that data will

be transferred through secure data platforms which may be outside the UK. We will only upload data in a format in which you cannot be identified.
All research data and blood samples will be labelled with a unique study number so that no-one that is not part of the research team will be able to identify you. The research team for the study will be able to link, confidentially, this unique number with your hospital records.

HOW WILL WE USE INFORMATION ABOUT YOU?
We will need to use information from you, from your medical records and your GP for this research project.

This information will include your:

- Initials
- NHS number
- Name
- Medical information

The information will be held by the study team at the Liverpool University Hospitals NHS Foundation Trust. The research records and the information may be used to make sure that the research is being done according to all regulations.

WHAT ARE YOUR CHOICES ABOUT HOW YOUR INFORMATION IS USED?

You can stop taking part in the study at any time, without giving a reason. We will ask you if we can keep information about you that has already been collected. If you do not wish this to happen, we will destroy all data and samples obtained so far.

If you choose to stop taking part in the study, we will ask you if we can continue to obtain information from your electronic medical record or your GP record. If you would not wish this to happen, please let us know and we will ensure we do not collect this information.

We need to manage your records in specific ways for the research to be reliable. This means that the data available in research files may be looked at by the study sponsor and regulatory authorities.

If you agree to take part in this study, we will ask you if you agree to have your data and/or samples used for future research projects. If you decide to withdraw from the study, we will not use your data and/or samples for any future research. However, the information, data and samples already collected as part of NOTE-AF will still be used as part of the results of the NOTE-AF trial. We need to manage your records in specific ways for the research to be reliable. This means that we won’t be able to let you see or change the data we hold about you.

At the end of the research study, it will be written up and submitted for publication in a medical journal and presented at academic meetings and conferences. You will not be identified in any published results.

We may also contact you to invite you for future studies. If you do not wish to take part in future research, please us know and we will ensure that we do not contact you.

WHERE CAN YOU FIND OUT MORE INFORMATION ABOUT YOUR INFORMATION WILL BE USED?
You can find out more about how we use your information from the following resources.

- The Health Research Authority www.hra.nhs.uk/information-about-patients/
- Our leaflet available from www.hra.nhs.uk/patientdataandresearch
- By asking one of the research team
- By sending an email to DPO@liverpoolft.nhs.uk
- By ringing us on 0151 706 2410

WHAT HAPPENS IF I LOSE CAPACITY?
There is a section on the consent form to provide details of a family member or friend who we may contact in this event that you lose the capacity to give an opinion on your behalf. You can nominate someone who knows your wishes and that you believe will act in your best interests. We will then contact this nominated contact to ask them if you would want to continue taking part in research.

WHAT WILL HAPPEN TO THE RESULTS OF THE RESEARCH?
After all data have been analysed, the results will be published in peer reviewed medical journals. It will not be possible to identify you specifically in any of the results.
NOTE-AF is part of a large European Research project called TARGET. When the study is completed, the data and files related to the study will be stored on a secure and password-protected data platform as part of the TARGET project. This is in line with European and UK data regulations. We will publish the results on the TARGET webpage (www.Target-Horizon.eu).

WHO IS ORGANISING AND FUNDING THE STUDY.

NOTE-AF is part of a large European Research project called TARGET. TARGET aims to develop tools to better treat AF and its complications, mainly stroke. It is being organised by a group of doctors and scientists in different countries across Europe. NOTE-AF is part of the project and led by Professor Ingeborg Welters, who is a consultant in Intensive Care Medicine at the Royal Liverpool Hospital. The study is funded by European Union, Horizon 2023. The sponsor of the study is Liverpool University Hospitals NHS Foundation Trust. The NHS indemnity scheme applies to provide insurance and/ or indemnity to meet the potential legal liability of the sponsor or employer for harm to participants.

WHO HAS REVIEWED THE STUDY?

All research in the NHS is looked at by an independent group of people, called a Research Ethics Committee, to protect your interests. This study has been reviewed and given a favourable opinion by *North West-Haydock Research Ethics Committee.*

Thank you for taking the time to read this information.

For more information about the NOTE-AF Study please contact:

Professor I. Welters

Tel:0151 706 3191

Email: i.welters@liverpoolft.nhs.uk

Dr. Hani Essa

Hani.essa@liverpoolft.nhs.uk

Dr. Brian Johnston

Brian.johnston2@liverpoolft.nhs.uk

Research Nurse Coordinator leading the study:

Karen.williams@liverpoolft.nhs.uk

0151 706 2410

If you are unhappy with any aspect of the study:

If you do not wish to speak to the research staff listed above, please contact:

Patient Advice and Complaints Team (PACTS)

Foyer of Royal Liverpool University Hospital

Prescot Street

Liverpool, L7 8XP

0151 706 2380

Email: PACT@liverpoolft.nhs.uk
