# Supplementary material for: Intelligent monitoring to predict atrial fibrillation (NOTE-AF): clinical study 1 for the ‘Health virtual twins for the personalised management of stroke related to atrial fibrillation (TARGET)’ project – a protocol for a prospective cohort analysis
Source: BMJ Open. 2026 Jan 3;16(1):e099658. doi: 10.1136/bmjopen-2025-099658 (PMC12766758; doi:10.1136/bmjopen-2025-099658)
Supplement: online supplemental file 2 [file bmjopen-16-1-s002.pdf]

IRAS ID-342528

Study Number:

Participant ID:	

PARTICIPANT CONSENT FORM

Intelligent Monitoring to Predict Atrial Fibrillation [NOTE-AF]: Clinical study 1 for the "Health virtual twins for the personalised management of stroke related to atrial fibrillation (TARGET)" project.

Name of Researchers: Professor I Welters, Dr. Hani Essa and Dr B Johnston

Please initial box

1. I confirm that I have read and understood the information sheet dated version for the above study. I have had the opportunity to consider the information, ask questions and have had these answered satisfactorily. ☐

2. I understand that my participation is voluntary and that I am free to withdraw at any time without giving any reason, without my medical care or legal rights being affected. ☐

3. I understand that relevant sections of my medical notes and data collected during the study, may be looked at by responsible individuals from Liverpool University Hospitals NHS Foundation Trust and regulatory authorities, where it is relevant to my taking part in this research.
I give permission for these individuals. to have access to my records. I understand that my personal details will be kept confidential. ☐

4. I understand that the information held and maintained by Liverpool University Hospitals NHS Trust may be used to provide information about my health status at Day 90. ☐

5. *I* understand that data collected about me during the study will be converted to anonymised data ☐

and transferred to a secure data platform within the UK or the European Union in a non-identifiable form.

6. I agree to my hospital Consultant team and General Practitioner being informed of my participation in the study. I agree to the hospital Consultant and GP being contacted during the study, and for any necessary exchange of information between them and the research team. ☐

7. I agree to take part in the above study. ☐

The following sections are optional *

8. I agree to surplus blood taken during routine care to be used as part of the research study.* *YES* ☐ *NO* ☐

*9.*I agree to be contacted for future research projects* *YES* ☐ *NO* ☐

*10.*I agree to my anonymised data and/ or blood samples being used in future studies.* *YES* ☐ *NO* ☐

*11. *I agree to take part in the WARD247 survey which is part of the above study.* *YES* ☐ *NO* ☐

Name of Participant	Date	Signature
___________________________	___________________________	__________________________

Name of Person taking consent Date Signature

(If different from researcher)

___________________________ ___________________________ ___________________________

Witness statement - for those mentally capable but physically unable to sign consent)

I hereby confirm that _____________________________was fully informed of the study as detailed in the information sheet and that informed consent was freely given

Name of patient (PRINT NAME)

.

_______________________________________ _________________ _______________________________

Witness (PRINT NAME) Date Signature

_______________________________________ _______________________________

Designation.

Nominated contact:

I understand that I am currently unwell and that my ability to answer questions and discuss this research may change during my treatment, if I am unable to answer for myself, I would like you to discuss my continued involvement in this study with

____

Their relationship to me is: -____

The best way to contact them is: - ____

When completed: 1 (original) to be kept in researcher site file, 1 for consultee; 1 for care record
