# Supplementary material for: Intelligent monitoring to predict atrial fibrillation (NOTE-AF): clinical study 1 for the ‘Health virtual twins for the personalised management of stroke related to atrial fibrillation (TARGET)’ project – a protocol for a prospective cohort analysis
Source: BMJ Open. 2026 Jan 3;16(1):e099658. doi: 10.1136/bmjopen-2025-099658 (PMC12766758; doi:10.1136/bmjopen-2025-099658)
Supplement: online supplemental file 4 [file bmjopen-16-1-s004.pdf]

IRAS ID-342528

Study Number:

Participant ID:	

PARTICIPANT CONSENT FORM REGAINED CAPACITY

Intelligent Monitoring to Predict Atrial Fibrillation [NOTE-AF]: Clinical study 1 for the "Health virtual twins for the personalised management of stroke related to atrial fibrillation (TARGET)" project.

Name of Researchers: Professor I Welters, Dr. Hani Essa and Dr Brian Johnston

Please initial box

1. I confirm that I have read and understood the information sheet dated _________________for the above study. I have had the opportunity to consider the information, ask questions and have had these answered satisfactorily. ☐

2. I understand that my participation is voluntary and that I am free to withdraw at any time without giving any reason, without my medical care or legal rights being affected. ☐

3. I understand that relevant sections of my medical notes and data collected during the study, may be looked at by responsible individuals from Liverpool University Hospitals NHS Foundation Trust and from regulatory authorities where it is relevant to my taking part in this research. I give permission for these individuals to have access to my records. I understand that my personal details will be kept confidential. ☐

4. I understand that the information held and maintained by Liverpool University Hospitals NHS Trust may be used to provide information about my health status at Day 90. ☐

5. *I* understand that data collected about me during the study will be converted to anonymised data and transferred to a secure data platform within the UK or the European Union in a non-identifiable form. ☐

When completed: 1 (original) to be kept in researcher site file, 1 for consultee; 1 for care record

6. I agree to my hospital Consultant team and General Practitioner being informed of my participation the study. I agree to my hospital Consultant team and my General Practitioner being contacted during the study and for any necessary exchange of information about me between them and the research team. ☐

7. I agree to continued participation in the above study. ☐

The following sections are optional *

8. ** I agree to surplus blood taken during routine care to be used as part of the research study* *YES* ☐

NO ☐

9. **I agree to be contacted for future research projects* ☐ ☐

10. *.*I agree to my anonymised data and/ or blood samples being stored for use in future studies* ☐ ☐

11. **I agree to take part in the WARD247 survey which is part of the above study.* ☐ ☐

Name of Participant	Date	Signature
___________________________	___________________________	___________________________

When completed: 1 (original) to be kept in researcher site file, 1 for consultee; 1 for care record

Name of Person taking consent Date Signature

(If different from researcher)

___________________________ ___________________________ ___________________________

Witness statement - for those mentally capable but physically unable to sign consent)

I hereby confirm that ___________________________
Name of patient (PRINT NAME)

was fully informed of the study as detailed in the information sheet and that informed consent was freely given.

_______________________________________ _________________ _______________________________

Witness (PRINT NAME) Date Signature

_______________________________________ _______________________________

Designation

When completed: 1 (original) to be kept in researcher site file, 1 for consultee; 1 for care record
