# Supplementary material for: Intelligent monitoring to predict atrial fibrillation (NOTE-AF): clinical study 1 for the ‘Health virtual twins for the personalised management of stroke related to atrial fibrillation (TARGET)’ project – a protocol for a prospective cohort analysis
Source: BMJ Open. 2026 Jan 3;16(1):e099658. doi: 10.1136/bmjopen-2025-099658 (PMC12766758; doi:10.1136/bmjopen-2025-099658)
Supplement: Supplementary file 5 [file bmjopen-2025-099658.reviewer_comments.pdf]

PEER REVIEW HISTORY

BMJ Open publishes all reviews undertaken for accepted manuscripts. Reviewers are asked to complete a checklist review form and are provided with free text boxes to elaborate on their assessment. These free text comments are reproduced below.

ARTICLE DETAILS

Title (Provisional)

INtelligent MOniToring to PrEdict Atrial Fibrillation [NOTE-AF]: Clinical study 1 for the “Health virtual twins for the personalised management of stroke related to atrial fibrillation (TARGET)” project. A protocol for a prospective cohort analysis.

Authors

Essa, Hani; Johnston, Brian; Lip, Gregory YH; Ortega-Martorell, Sandra; Williams, Karen; Welters, Ingeborg; Consortium, TARGET

VERSION 1 - REVIEW

**Reviewer**	**1**
**Name**	**Wong, Kam Cheong**
**Affiliation**	**The University of Sydney Faculty of Medicine and Health, Westmead Applied Research Centre**
**Date**	**15-Feb-2025**
**COI**	**None**

Atrial fibrillation (AF) prevalence is on the rise, and risk prediction tools are used to guide clinical decisions in treating patients with AF. It is timely that the study aims to address the limitations of the current risk prediction tools, such as CHA2DS2-VASc, CHA2DS2-VA, which are based on static risk factors. It is hypothesised that the risks of AF development were associated with dynamic factors, including acute illnesses and some perioperative surgical procedures. The authors aim to examine and establish an AF prediction tool for use in specific dynamic circumstances. They will apply various quantitative and qualitative methods to assess and identify pathophysiological and clinical factors that could predict AF in such circumstances, including examining the acceptability and usability of their approach using qualitative evaluation.

However, I have a few concerns about some of the methods.

1. One of the aims/outcomes is to examine the incidence of post-operative AF. Pre-operative ECG assessment is common. Will pre-operative ECGs be included in the analysis (e.g. presence of baseline premature atrial complexes (PAC), AV blocks, etc)? How will they be analysed? (Note: the authors aim to examine PACs as

precursors of AF. Thus, examining the presence of pre-operative PAC may be relevant.)

2. A wireless continuous vital sign monitoring system (wCVSM) will be used. The authors declare that this system will pose major challenges to the routines of nurses and doctors, e.g., affecting how nurses respond to patients and how they call for action (see the submission page 51/120). Is there a response plan to help nurses and doctors make decisions? The response plan could be added as a supplementary document, which would be helpful to readers.
3. A seven-day monitor has been planned. Why seven days? It would be more convincing if you could back this up with reasons and citations/ literature.
4. Length of stay and 30- and 90-day readmission rates will be included as secondary outcomes. Is it possible to include the "primary reason for admission/ readmission" in the analysis? This would shed light on AF-related admissions and readmissions.
5. In addition to the wCVSM, the analysis will include in-hospital investigations such as biomarkers (troponin, natriuretic peptides and inflammatory markers) and echocardiography. The indications for these investigations could vary among patients. Would it be relevant and possible to include the "indications for investigations" alongside these parameters? They may provide insight into the clinical indications of the patients.
6. Although the statistical analysis section has outlined the fundamental statistical methodology, a Statistical Analysis Plan (SAP) as a supplement document is lacking. An SAP would have included the details of the planned analysis, including how the above-mentioned outcomes and parameters would fit into various analyses.
7. The current protocol lacks details on the qualitative methodology used to assess acceptability and usability. There are too many closed questions, i.e., six out of eight interview questions are "closed questions", which could limit the depth of response (see the submission page 72/120). More importantly, the theoretical underpinning for formulating the questions is not specified. Specifying the theoretical underpinning is one of the requirements for qualitative evaluation (see COREQ guideline, https://doi.org/10.1093/intqhc/mzm042). For example, applying Critical Realism to underpin the interview formulation and interpretation (https://doi.org/10.4324/9780203843314) and using the UK Medical Research Council Guidance of Process Evaluation to guide the evaluation framework (*doi:10.1136/bmj.h1258).* The qualitative evaluation potentially identifies strengths, weaknesses, opportunities and threats, which could be analysed using SWOT analysis to inform policy and guideline developments. The authors may reference an example publication incorporating Critical Realism, the UK Medical Research Council Guidance of Process Evaluation and SWOT analysis: https://doi.org/10.1136/bmjopen-2024-088260.

I suggest revising the manuscript.

**Reviewer**	**2**
**Name**	**Uzięblo-Życzkowska, Beata**
**Affiliation**	**Military Institute of Medicine - National Research Institute, Department of Cardiology and Internal Diseases**
**Date**	**26-Jul-2025**
**COI**	**None**

I strongly recommend this protocol of the NOTE-AF study for publication in BMJ Open. The topic addressed in the study is highly clinically relevant and worth investigating. The study rationale, methodology, and inclusion and exclusion criteria have been properly formulated. The chosen statistical methods are appropriate for the stated objectives.

The study results will undoubtedly be of interest and will fill a gap in the current data regarding patients with atrial fibrillation.

VERSION 1 - AUTHOR RESPONSE

Reviewer: 1
Dr. Kam Cheong Wong, The University of Sydney Faculty of Medicine and Health, The University of Sydney Faculty of Medicine and Health
Comments to the Author:
Atrial fibrillation (AF) prevalence is on the rise, and risk prediction tools are used to guide clinical decisions in treating patients with AF. It is timely that the study aims to address the limitations of the current risk prediction tools, such as CHA2DS2-VASc, CHA2DS2-VA, which are based on static risk factors. It is hypothesised that the risks of AF development were associated with dynamic factors, including acute illnesses and some perioperative surgical procedures. The authors aim to examine and establish an AF prediction tool for use in specific dynamic circumstances. They will apply various quantitative and qualitative methods to assess and identify pathophysiological and clinical factors that could predict AF in such circumstances, including examining the acceptability and usability of their approach using qualitative evaluation.

However, I have a few concerns about some of the methods.
1. One of the aims/outcomes is to examine the incidence of post-operative AF. Pre-operative ECG assessment is common. Will pre-operative ECGs be included in the analysis (e.g. presence of baseline premature atrial complexes (PAC), AV blocks, etc)? How will they be analysed? (Note: the authors aim to examine PACs as precursors of AF. Thus, examining the presence of pre-operative PAC may be relevant.)

>>>Our study comprises different patient cohorts, not all patients will have routine ECGs available before the onset of their acute condition. Where available as part of standard care, findings of previous ECGs will be collected as baseline characteristics, including but not

limited to PR interval, P wave dispersion, AV block, resting heart rate and branch block. We have now displayed the main baseline characteristics in a table (1). We believe that for assessment of the predictive value of PACs continuous ECG monitoring, which forms a major part of the study intervention, is key.

See page 11

2. A wireless continuous vital sign monitoring system (wCVSM) will be used. The authors declare that this system will pose major challenges to the routines of nurses and doctors, e.g., affecting how nurses respond to patients and how they call for action (see the submission page 51/120). Is there a response plan to help nurses and doctors make decisions? The response plan could be added as a supplementary document, which would be helpful to readers.

In line with the primary research objective (To determine the incidence of clinical and subclinical episodes of AF in acutely unwell patients and to generate data for the development and validation of virtual twins and clinical decision support tools) wCVSM will run in parallel with standard of care. No decision making will be based on the data obtained, unless the attending healthcare professional decides to do so. This is possible as all monitoring systems are CE-marked medical devices. In case of alerts indicating clinical deterioration and after confirmation by healthcare professionals, standard therapy pathways for the treatment of the deteriorating patients will be followed. We have added the following sentence into the protocol: "All patients will continue to receive standard routine care in addition to wCVSM. Standard clinical treatment will be provided in line with the organisation's policies for monitoring of vital signs. In case of clinical deterioration, standard treatment pathways will be followed."

Please see page 11

3. A seven-day monitor has been planned. Why seven days? It would be more convincing if you could back this up with reasons and citations/ literature.

>>>We agree that explaining the rationale would be useful. We believe that the literature backs up the viewpoint that most episodes of new-onset AF occur early on during a patient's illness/hospitalisation, but early recurrence is common and may not be detected without

extending the monitoring period. We have added the below paragraph in the discussion to explain this further:

"It is well documented that in acutely unwell patients, the majority cases of new-onset AF (NOAF) occur within the first 3 days of admission[28 29]. This is because the burden of acute systemic stressors/triggers (hypoxia, electrolyte disturbance, cytokine surges and sympathetic activation) are often highest in the early phase of the disease. Prior work looking at patients suffering with septic shock demonstrated that episodes of NOAF usually occur prior to day 5[30]. Extending the monitoring period to 10 days will allow to collect information about recurrence and duration of episodes of NOAF in acute illness. Previous literature suggests that in patients hospitalized for noncardiac surgery or medical illness who had transient new-onset AF, recurrence occurs in one third of patients within one year. Similarly, recurrence of AF episodes in ICU patients who are treated successfully for their first episode, is also common.

See page 19

4. Length of stay and 30- and 90-day readmission rates will be included as secondary outcomes. Is it possible to include the "primary reason for admission/ readmission" in the analysis? This would shed light on AF-related admissions and readmissions.

Primary admission diagnosis as well as reasons for readmissions will be collected as routine standard of care and can be extracted from Electronic Health Records. We have added the following sentence to clarify this:

"The use of Electronic Patient Health Records is part of standard care at the recruiting site to document routine patient data and will allows extraction of standard data, including length of stay, admission diagnosis, data, time and reason for readmission, new diagnoses and clinically relevant cardiovascular events."

Please see page 17

5. In addition to the wCVSM, the analysis will include in-hospital investigations such as biomarkers (troponin, natriuretic peptides and inflammatory markers) and echocardiography. The indications for these investigations could vary among patients.

Would it be relevant and possible to include the "indications for investigations" alongside these parameters? They may provide insight into the clinical indications of the patients.

We agree that it is a relevant and important question to understand the indications for cardiac biomarker testing in acute care. As such we will record indications and clinical events associated with increases in cardiac biomarkers, however, it goes beyond the scope of the NOTE-AF study to systematically and prospectively assess the indications for requesting such investigations. We know from previous audits that significant variation in documenting the indications for cardiac biomarkers exist. Since we will use standard data and surplus blood samples, we will only be able to draw associations between biomarkers and clinical events as part of a follow-up retrospective analysis, which will be described in a separate protocol. The details of this protocol will depend on the number and time points of surplus samples and routine troponin and BNP measures being available.

6. Although the statistical analysis section has outlined the fundamental statistical methodology, a Statistical Analysis Plan (SAP) as a supplement document is lacking. An SAP would have included the details of the planned analysis, including how the above-mentioned outcomes and parameters would fit into various analyses.

We appreciate the reviewer's observation regarding the a detailed Statistical Analysis Plan (SAP) in the current document.

The primary research objective for NOTE-AF is "To determine the incidence of clinical and subclinical episodes of AF in acutely unwell patients and to generate data for the development and validation of virtual twins and clinical decision support tools." In order to address the primary objective, novel models for prediction and risk assessment for new episodes of AF in acutely ill patients will be developed using machine learning and novel virtual health technology. These developments form distinct Work Packages within TARGET and will be published separately. Publications will be made available online (https://target-horizon.eu/resources/). In line with this approach and as stated in the document, conventional statistical analysis will be limited to descriptive statistics and univariate analysis between patients who develop episodes of AF and those who do not.

This approach ensures transparency and methodological rigour, while maintaining flexibility to adapt the plan to evolving data structure and quality, in line with best practices for observational studies embedded in large consortia projects"

7. The current protocol lacks details on the qualitative methodology used to assess acceptability and usability. There are too many closed questions, i.e., six out of eight interview questions are "closed questions", which could limit the depth of response (see the submission page 72/120). More importantly, the theoretical underpinning for formulating the questions is not specified. Specifying the theoretical underpinning is one of the requirements for qualitative evaluation (see COREQ guideline, https://doi.org/10.1093/intqhc/mzm042). For example, applying Critical Realism to underpin the interview formulation and interpretation (https://doi.org/10.4324/9780203843314) and using the UK Medical Research Council Guidance of Process Evaluation to guide the evaluation framework (doi:10.1136/bmj.h1258). The qualitative evaluation potentially identifies strengths, weaknesses, opportunities and threats, which could be analysed using SWOT analysis to inform policy and guideline developments. The authors may reference an example publication incorporating Critical Realism, the UK Medical Research Council Guidance of Process Evaluation and SWOT analysis: https://doi.org/10.1136/bmjopen-2024-088260.

We thank the reviewer for these valuable comments. However, we wish to point out that the full development of a qualitative study protocol, taking into account the guidelines and concepts mentioned above, goes well beyond the scope of this manuscript. We have further specified the structure of the NOTE-AF protocol in the introduction as follows:

"The NOTE-AF clinical study has distinct primary and secondary objectives that are detailed further in the methods section. The two work packages (WP) comprising the NOTE-AF study are linked to the TARGET Project, with WP1 representing the observational part of NOTE-AF, which is described in this manuscript."

Acceptability and usability are not fully assessed in this study. We will take the opportunity for preliminary data collection, aiming to develop a feasibility study followed by an implementation study or a process evaluation, which will require a full study protocol taking into account the points mentioned above (COREQ guideline, UK Medical Research Council Guidance of Process Evaluation and SWOT analysis). We have therefore focused on the observational part, pointing out that the usability and acceptability assessment will be published in more detail elsewhere. This approach will also allow comparison and a standardised approach between international healthcare settings as part of a wider international study. In line with Health Authority Regulations within the UK, description of the qualitative data, the MUAQ questionnaire and the semi-structured interview template is required to obtain Health Research Authority and Research Ethics Committee approval.

Reviewer: 2

Dr. Beata Uziębło-Życzkowska, Military Institute of Medicine - National Research Institute

Comments to the Author:

I strongly recommend this protocol of the NOTE-AF study for publication in BMJ Open. The

topic addressed in the study is highly clinically relevant and worth investigating. The study rationale, methodology, and inclusion and exclusion criteria have been properly formulated. The chosen statistical methods are appropriate for the stated objectives. The study results will undoubtedly be of interest and will fill a gap in the current data regarding patients with atrial fibrillation.

>>>We thank the reviewer for their kind comments above.

VERSION 2 - REVIEW

**Reviewer**	**1**
**Name**	**Wong, Kam Cheong**
**Affiliation**	**The University of Sydney Faculty of Medicine and Health, Westmead Applied Research Centre**
**Date**	**30-Aug-2025**
**COI**	

No further comment.
